# Expansion of CORE-SINEs in the genome of the Tasmanian devil

**DOI:** 10.1186/1471-2164-13-172

**Published:** 2012-05-06

**Authors:** Maria A Nilsson, Axel Janke, Elizabeth P Murchison, Zemin Ning, Björn M Hallström

**Affiliations:** 1 LOEWE - Biodiversity and Climate Research Center, BiK-F, Senckenberganlage 25, Frankfurt am Main, D-60325, Germany; 2 Wellcome Trust Sanger Institute, Hinxton, CB10 1SA, UK; 3 Goethe University Frankfurt, BioCampus, Siesmayerstr. 70, Frankfurt am Main, 60323, Germany

**Keywords:** SINE, WSINE1, Retroposon, Tasmanian devil, Sarcophilus, Genome, Marsupials

## Abstract

**Background:**

The genome of the carnivorous marsupial, the Tasmanian devil (*Sarcophilus harrisii*, Order: Dasyuromorphia), was sequenced in the hopes of finding a cure for or gaining a better understanding of the contagious devil facial tumor disease that is threatening the species’ survival. To better understand the Tasmanian devil genome, we screened it for transposable elements and investigated the dynamics of short interspersed **e**lement (SINE) retroposons.

**Results:**

The temporal history of Tasmanian devil SINEs, elucidated using a transposition in transposition analysis, indicates that WSINE1, a CORE-SINE present in around 200,000 copies, is the most recently active element. Moreover, we discovered a new subtype of WSINE1 (WSINE1b) that comprises at least 90% of all Tasmanian devil WSINE1s. The frequencies of WSINE1 subtypes differ in the genomes of two of the other Australian marsupial orders. A co-segregation analysis indicated that at least 66 subfamilies of WSINE1 evolved during the evolution of Dasyuromorphia. Using a substitution rate derived from WSINE1 insertions, the ages of the subfamilies were estimated and correlated with a newly established phylogeny of Dasyuromorphia. Phylogenetic analyses and divergence time estimates of mitochondrial genome data indicate a rapid radiation of the Tasmanian devil and the closest relative the quolls (*Dasyurus*) around 14 million years ago.

**Conclusions:**

The radiation and abundance of CORE-SINEs in marsupial genomes indicates that they may be a major player in the evolution of marsupials. It is evident that the early phases of evolution of the carnivorous marsupial order Dasyuromorphia was characterized by a burst of SINE activity. A correlation between a speciation event and a major burst of retroposon activity is for the first time shown in a marsupial genome.

## Background

The Tasmanian devil (*Sarcophilus harrissii*) is the largest living carnivorous Australian marsupial in the order Dasyuromorphia, which also includes the anteating numbat, quolls, dunnarts, phascogales, and the iconic extinct Tasmanian tiger (*Thylacinus cynocephalus*) [1]. Populations of the Tasmanian devil are currently under serious threat of being severely diminished or eradicated by devil facial tumor disease (DFTD), a contagious form of cancer [2,3]. Since it was first observed in 1996, DFTD has nearly halved the Tasmanian devil population [3,4]. Without a cure for DFTD, Tasmanian devils may become extinct in 10–20 years [4,5]. However, with the help of next generation sequencing techniques, finding a possible cure may be within reach [6]. To gain a better understanding of the genetics of the disease and possibly find a cure, the genome of the Tasmanian devil was recently sequenced [7,8].

Recent studies have shown that marsupial genomes have been under massive bombardment by transposable elements [9-11], which play important roles in genomic evolution [12-14] and are the causes of some diseases [15,16]. In the opossum, 52% of the genome sequence consists of such elements. This is the largest percentage of transposable elements presently identified in any vertebrate genome. In comparison, the human genome consists of 45% and the mouse genome of 38% transposable elements [17,18]. Transposable elements are classified as retroposons, which propagate via an RNA-intermediate, and DNA transposons that transpose themselves directly by DNA, without an intermediate [19]. Typically, transposons make up only a small percentage of genome sequences, while the vast majority of the transposable elements are retroposons [19]. One group of retroposons, the non-autonomous SINEs (**s**hort **in**terspersed **e**lements), is propagated in the genome via the activity of the autonomous LINEs (**l**ong **in**terspersed **e**lements) [19-][21].

Most SINEs originate from tRNAs and their basic structure includes a 5′ tRNA-related sequence, a tRNA unrelated body, a 3′ LINE-related tail sequence that sometimes is followed by a poly(A)- stretch [19]. The 3′ tail sequence is derived from LINEs and is used for recognition by the reverse transcriptase. The marsupial SINEs have been shown to have tail sequences originating from LINE2, LINE3 as well as RTE elements [22]. When a polyA stretch is present in the 3′ end of the SINE it is used by LINE1 for retroposition [23]. A group of highly conserved SINEs in mammals are the CORE-SINEs. CORE-SINEs have a specific and highly conserved 65‒nt central sequence between the tRNA-related part and the LINE derived region [24]. CORE-SINEs have been found in diverse groups of animals, such as mammals, mollusks and fish [24-27]. WSINE1, a characteristic SINE of marsupials, is a short CORE-SINE, consisting only of the tRNA-related part and a truncated 41-nt CORE sequence followed by a poly(A)tail [28]. In placental mammals, the CORE-SINEs have been inactive fossils for at least 130 million years (Myr) [26]. However, this retroposon type has remained active in marsupial and monotreme genomes. In monotreme genomes, the MON CORE-SINE dominates [29], and in marsupials, the CORE-SINEs have remained active and have proliferated, giving rise to many different subtypes [10,11,30].

While most placental mammalian orders are currently represented by at least one genome sequence, genomic sequences of only three marsupial species, representing three of the seven orders, are currently known: the South American opossum (*Monodelphis domestica*) [11], the Australian wallaby (*Macropus eugenii*) [31], and the Tasmanian devil. Molecular data show that the respective orders of the first two, Didelphimorphia (*Monodelphis*) and Diprotodontia (*Macropus*), are separated by 75–65 Myr of evolution [32,33], representing the geographically and temporally deepest divergence among marsupials. The retroposon content in the opossum genome has been thoroughly investigated [9,10], and here we have screened the genome of an Australian marsupial to understand the dynamics of SINEs and non-autonomous LINEs. The Tasmanian devil genome sequence will be a valuable source for understanding the dynamics of retroposons in marsupial genomes.

## Results

### Full genome screening

**Figure 1:**
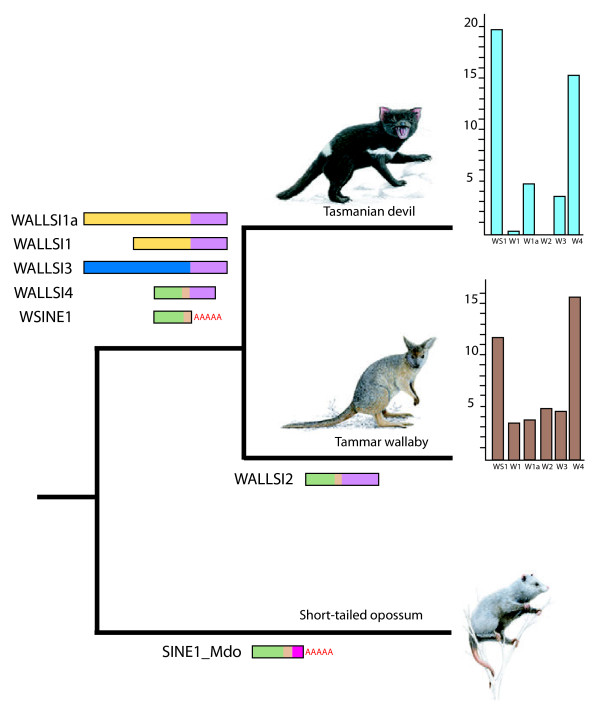
**Distributions of CORE-SINEs and the RTE-propagated WALLSIs across Australian marsupials.** The horizontal diagrams represent schematic retroposon structures. Light green: tRNA CORE-SINE with a beige CORE sequence, red As: poly(A)-tail for propagation via LINE1, purple: RTE-tail, yellow, dark blue, and pink: retroposon-specific sequences. In the plots to the right, each bar indicates numbers of copies in 10,000s. WS1:WSINE1; W1:WALLSI1; W1a: WALLSI1a; W2: WALLSI2; W3: WALLSI3; W4:WALLSI4.

A summary of the abundances of all transposable elements revealed in the *in silico* screening of *Sarcophilus harrisii* is shown in Additional file 1: Table S1. The most abundant marsupial specific SINEs and non-autonomous LINEs were calculated (Table 1; Figure 1).

**Table 1:** Abundances of selected SINEs and non-autonomous LINEs in the Tasmanian devil and wallaby genomes

SINE type	Total nts Tas. devil	Copy- number Tas. devil	% genome Tas. devil	Copy- number wallaby	% genome wallaby
WSINE1	21214630	198314	0.72%	128223	0.66%
WALLSI2	0	0	0%	48143	0,42%
Mar1a_Mdo	29386582	143332	1.00%	131108	0.86%
Mar1b_Mdo	31618046	208863	1.08%	176658	1.00%
WALLSI3	8715448	36908	0.30%	44063	0.08%
WALLSI1	190557	1877	0.01%	32754	0.30%
WALLSI1a	10610371	47889	0.36%	36886	0.30%
MAR1	2938652	22539	0.10%	25150	0.09%
Mar1c_Mdo	6500819	50970	0.22%	39736	0.20%
P7SL_MD*	2408356	12942	0.08%	12720	0.10%
WALLSI4	21363683	153231	0.73%	154833	0.91%
MdoRep1	14913384	119520	0.51%	124233	0.62%
MIR3/THER-2	79246143	678295	2.71%	715973	3.44%
MIR/THER-1	66889878	594020	2.28%	490584	2.15%

* A putative pseudogene

### Transposition in Transposition

**Figure 2:**
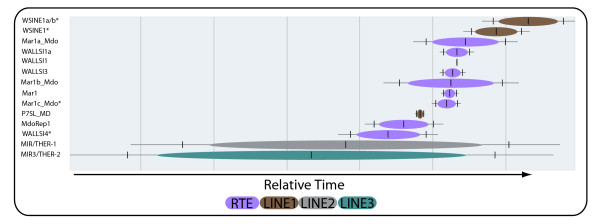
**SINE Transposition in Transposition.** TinT (Transposition in Transposition) of the most abundant marsupial-specific SINEs and non-autonomous LINEs in the Tasmanian devil genome. The chronological order of SINE activity goes from bottom (oldest) to top (most recent), with WSINE1 representing the most recently active SINE. The length of the bars indicates the length of time a SINE was active, with MIR3/Ther-2 having the longest activity period and P7SL_MD and WALLSI1 the shortest. The color-coding indicates the types of elements responsible for the respective SINE retropositions. The * indicates truncation of the CORE sequence.

For comparative genomics and in particular evolutionary purposes, it is useful to be able to estimate the relative order of activities of different groups of integrated SINEs found in the genome. TinT (transposition in transposition) is an algorithm that screens the genome for nested retroposon insertions (i.e., retroposons inserted within another retroposon) and calculates its relative time frame of activity based on the fact that old inactive retroposons cannot insert into younger active retroposons [34]. Screening of the Tasmanian devil genome with the TinT program [34] revealed 17,928 nested insertions for the most frequent SINEs and non-autonomous LINEs (Figure 2) ( Additional file 1: Table S2). The most recently active element is the WSINE1. The TinT pattern from the Tasmanian devil is reasonably similar to those previously observed in the opossum and wallaby [35].

### WALLSI elements

Several WALLSIs (**Wall**aby **SI**NEs) have been described in the wallaby genome [36-40]. Three types, the WALLSI1/1a, WALLSI2, and WALLSI3, have highly similar tail regions derived from RTE_MD (70% similarity). WALLSI2 and WALLSI4 are both CORE-SINEs. Except for WALLSI2, we found all previously identified WALLSIs in the Tasmanian devil genome but in different proportions than previously described (Table 1, Figure 1). There are fewer copies of the 5′-truncated WALLSI1 in the Tasmanian devil (assembly size 2984.5 Mbp) (1,877 WALLSI1) than in the wallaby (assembly size 2591.3 Mbp) (nearly 32,754). By contrast, the 200-nt-longer WALLSI1a is present in 47,889 copies (10.6 Mbp total—16 copies/Mbp) in the Tasmanian devil genome, which is more than the 36,886 (7,9 Mbp total—14 copies/Mbp) copies in the current assembly of the wallaby genome, which might be an underestimate given the low coverage of the genome sequence. On the other hand WALLSI4 is present in relatively similar numbers in both genomes (153,231 vs. 154,833) (Table 1), which might indicate that the activities of the two elements terminated before the two orders split 55–60 Myr ago and the SINEs were passed on to the descendants. The WALLSI3 is more abundant in the wallaby genome (36,908 vs. 44,063) despite lower sequence coverage of the assembled genome suggesting an expansion of the element. We did not identify any copies of WALLSI2 in the Tasmanian devil genome by the *in-silico* approach. WALLSI2 was initially believed to be restricted to kangaroos (under the name Mac1 [30]); however, they have also been identified in other diprotodontian marsupials [35], indicating that the element is not restricted to the kangaroo subfamily Macropodidae, but rather is characteristic of the entire order Diprotodontia.

### PSL7_MD

Another interesting feature of the marsupial genome is the P7SL_MD retropseudogene [9]. The cytoplasmic 7SL RNA is part of a signal recognition particle, and around 60 Myr ago it formed the Alu element, which is the most abundant SINE in primates [41] and other related SINEs (B1, Tu-type) in the super-order Euarchontoglires [41,42]. However, Alus are imperfect dimers derived from the 7SL sequence. In marsupials, the P7SL_MD retropseudogene sequence is very similar to the 7SL RNA. It is present in nearly identical copy numbers in the genomes of opossum [9], wallaby [35], and Tasmanian devil, where we discovered more than 12,000 copies. The high abundance may indicate that this pseudogene has undergone an extreme expansion in the ancient marsupial genome that has not been observed in any other mammal.

### WSINE1

The WSINE1 family is the most abundant of the recently active SINEs in the Tasmanian devil. We identified approximately 200,000 WSINE1 elements, but even this may be an underestimate, as we also found nearly 90,000 copies of fragmented CORE-SINEs that may represent ancient and diverged copies of WSINE1. Due to the difficulty of correctly assigning these fragmented SINEs, they have not been included in the final estimates.

**Figure 3:**
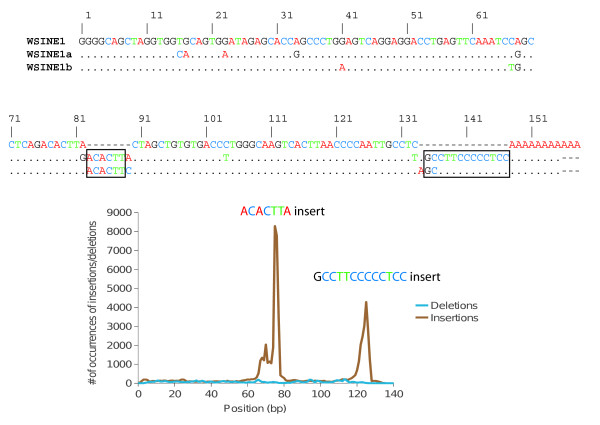
**Indel analysis of WSINE1.** Alignment of the three consensus sequences for the subtypes WSINE1, WSINE1a, and WSINE1b. Computational indel screening of the Tasmanian devil WSINE1s shows that two indels are over-represented, one insertion at position 80 and one at position 120. The smaller peak at position 70 is the result of an artifact in the automatic alignment process; when the last base of the apparent 7-bp insertion is substituted with an adenine (A), it preferentially aligns with the identical nucleotides upstream of the insertion site.

We also identified a third, novel subtype of WSINE1 in the Tasmanian devil genome that deviates from the previously described WSINE1s, WSINE1 and WSINE1a [28,43]. This new subtype, WSINE1b, lacks the 11-nt insert near the poly(A)-tail (pos. 120) found in WSINE1a elements. WSINE1a and WSINE1b both have a 7-nt insert at position 80, suggesting a close relationship. A detailed indel analysis of all WSINE1 elements shows that there are no other commonly occurring insertions or deletions (Figure 3). All three types of WSINE1 are present in the genome of the Tasmanian devil, but in different proportions. Nearly 90% belong to the new WSINE1b subtype, while the WSINE1a elements represent only 1.4% of all WSINE1s (Table 2).

**Table 2:** Distribution of WSINE1 variants in three different marsupial orders

Species	WSINE1	WSINE1a	WSINE1b
*Sarcophilus*	9.29% (7864)	1.4% (1187)	89.3% (75603)
*Isoodon*	0.1% (1)	3.0% (31)	96.9% (989)
*Macropus*	73.6% (23499)	7.7% (2463)	18.6% (5956)

The values for Tasmanian devil are based on the analysis of the full genome, *Isoodon* on the available trace sequences (approximate 5 million nt), and *Macropus* on a screening of the complete genome. The abundances of the three variants are expressed as % with the copy number in parenthesis. Only the complete WSINE1s (84,654 copies) were used in the analysis, as many are fragmented and lack the regions needed to safely define the subfamily.

### WSINE1 distribution in Australian marsupial orders

To examine the evolutionary dynamics of WSINE1 we also investigated the distributions of the WSINE1 subtypes in the genomes of two other marsupial orders, the Peramelemorphia (*Isoodon*; bandicoot) and Diprotodontia (*Macropus*; wallaby). The screened trace sequences of *Isoodon* show a pattern similar to that of the Tasmanian devil, with the majority of WSINEs belonging to the WSINE1b subtype. By contrast, in the wallaby genome the most abundant WSINE is the WSINE1 (73.6%), though WSINE1b is still widespread, making up almost 20% of the WSINE copies (Table 2).

### The age and number of the WSINE1 subfamilies

**Figure 4:**
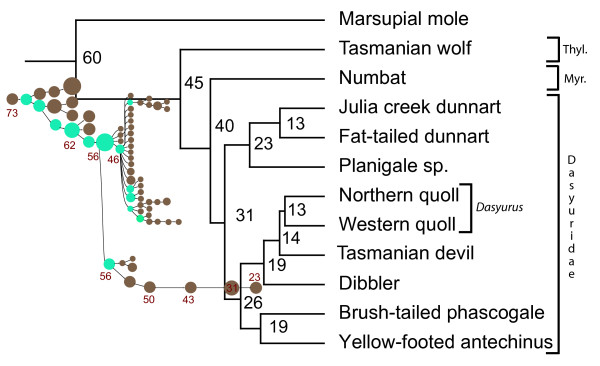
**Phylogeny of Dasyuromorphia based on complete mt genomes.** Phylogenomic relationships among dasyuromorphian subfamilies, including the position of the Tasmanian devil, based on complete mitochondrial genomes. The numbers in black at the nodes of the tree are divergence times calculated from a 44-taxon matrix using a combined set of 13 fossil calibration points. The circles plotted beside the tree are the results of a COSEG analysis of the 66 subfamilies of WSINE1. Brown circles indicate different subfamilies and blue circles the subfamilies that gave rise to new subfamilies. The red numbers beside the circles indicate the estimated evolutionary age for selected subfamilies. Thyl: Thylacinidae; Myr: Myrmecobiidae.

Co-segregation analysis (COSEG [44]) of 171,000 full-length WSINE1 elements identified 66 subfamilies in the Tasmanian devil genome, with copy numbers ranging from 500 to 9727 ( Additional file 1: Figure S1, Additional file 1: Table S3). The evolutionary age of each subfamily was determined using a local nucleotide substitution rate derived from WSINE1 ( Additional file 1: Table S4). The oldest elements were estimated to be 73 Myr old and the youngest 23 Myr old (Figure 4). It is evident that a burst of new subfamilies appeared around 56–46 Myr ago. Of these, only a handful continued to be active and to give rise to additional master copies. More than 20 new subfamilies were created during a very short time span. The burst of additional subfamilies occurred around the same time as the early evolutionary phases in the order Dasyuromorphia (Figure 4).

### Phylogenetic relationships and divergence times of the Tasmanian devil

We conducted mitochondrial genomic analyses to reconstruct the phylogenomic relationships of the Tasmanian devil to other carnivorous marsupials and to estimate the divergence times of this order. This allowed us to correlate the evolutionary age of Dasyuromorphia and place their early divergences in relation to the emergence of different WSINE subfamilies.

The mitogenomic analysis identified the Tasmanian devil to be the sister group to the quolls (*Dasyurus*; Figure 4). However, only after including third codon positions, in the nucleotide analysis could the Tasmanian devil be placed outside *Dasyurus*, indicating a rapid divergence of these lineages. As third codon positions are the fastest evolving nucleotide position only these seem to have acquired enough information during the short interval between the groups (*Dasyurus* and *Sarcophilus*) to resolve this relationship. It was equally difficult to estimate the relationships among the three subfamilies Thylacinidae, Myrmecobiidae, and Dasyuridae, as SH- and AU-tests did not reject alternative hypotheses, which is consistent with a rapid speciation process ( Additional file 1: Table S5).

The origin of the Tasmanian devil is estimated by both sets of calibration points to be 12–14 Myr ago ( Additional file 1: Table S6). The split within *Dasyurus* is estimated to be 13 Myr, merely 1 to 2 million years after the origin of the genus *Sarcophilus* (Tasmanian Devil). The deepest split within Dasyuridae is estimated to be 31 Myr ( Additional file 1: Figure S2). The origin of Dasyuromorphia is estimated to be 45 Myr, with a second split around 40 Myr leading to Numbat (Myrmecobiidae).

## Discussion

### Tasmanian devil genome-composition and activity of retroposons

*In silico* screening showed that transposable elements account for about 52.2% of the Tasmanian devil genome sequence ( Additional file 1: Table S1), which is virtually identical to that found in the opossum (52.2%) and wallaby genome (52.8%) [11,31]. The SINEs and non-autonomous LINEs make up 10.9% of the Tasmanian devil sequence (Table 1), which is comparable to the percentages identified in the genomes of the opossum (10.44%) and wallaby (11.7%) [11,31].

The relative temporal activity of SINEs was studied using the TinT program. When a younger retroposon integrates into another older retroposon, it is possible to estimate relative times of activity based on the information from these nested retroposition events [34]. As retroposons tend to be active in distinct waves, the temporal chain of activities can be calculated based on thousands of nested retroposition events found in the genome. The TinT method is a valuable tool for screening genomic data for phylogenetically informative retroposons, as the search can be targeted towards younger or older branches of the tree depending on the choice of elements. The method has been successfully applied to different groups of animals, such as birds [45], lagomorphs [46], primates [34], platypus [29], and marsupials [35]. Using the TinT approach, different SINEs and non-autonomous LINEs were investigated in the Tasmanian devil genome. Several of them (e.g., MIRs, Mar1a,b,c_Mdo, WALLSI4, WALLSI3 are shared among the three marsupials, wallaby, opossum, and Tasmanian devil (for which whole genome sequence data are available) with relatively similar activity patterns (Figure 2). Based on the TinT analysis, the element that had a recent expansion in the Tasmanian devil genome is the CORE-SINE of the type WSINE1.

### WSINE1

The WSINE1s are more abundant in the Tasmanian devil genome than in the wallaby genome (Table 1; Figure 1) and had the most recent expansion of all SINEs (Figure 2). The WSINE1 is a short element, consisting of a tRNA-derived region, a CORE-sequence region, and a poly(A)‒tail. The CORE-sequence region is extraordinarily conserved across mammals, which has led to speculations concerning functional properties of this region [47]. Additionally, the element has a poly(A)-tail and is flanked by target site duplications and is therefore expected to be LINE1 propagated [23,28]. In many of the marsupial CORE-SINEs the CORE-region is 41 nt long. In placental mammalian CORE-SINEs, however, the original CORE‒sequence is 65 nt long [24,27,30].

Two subtypes of WSINE1 were previously reported from studies of the wallaby genome: the WSINE1 and the WSINE1a [28,43]. After screening and comparative sequence analysis of the full-length WSINE1 in the Tasmanian devil genome, it was obvious that the majority of WSINE1s belongs to a third subfamily; 89% of the WSINE1s belong to what we refer here to as WSINE1b (Figure 3, Table 2).

**Figure 5:**
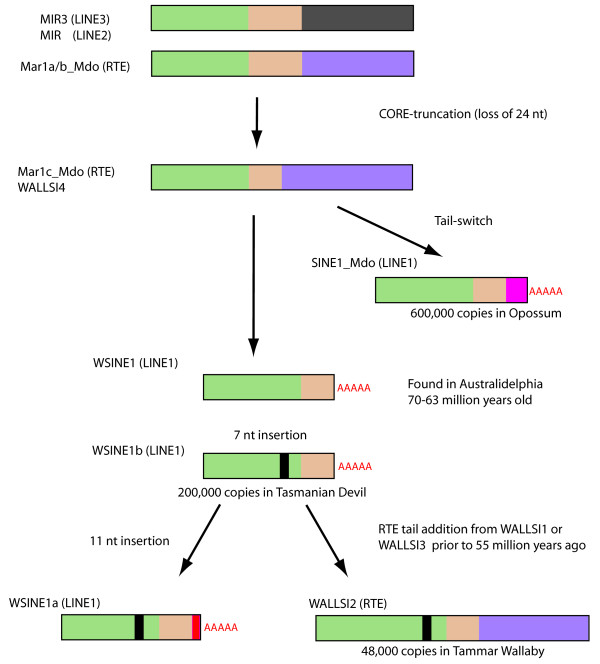
**Relationship among recent CORE-SINEs in marsupial genomes.** A hypothetical evolutionary scenario of the relationships among short CORE-SINEs, particularly WSINE1 and WALLSI2, found in marsupials. The short CORE (beige) is only found in a few SINEs, suggesting a common evolutionary origin. Insertions into the tRNA-related part (green) of CORE-SINEs are rare; thus, the 7-nt insert (black) shared between WSINE1a/1b and WALLSI2 is likely to have a common evolutionary origin. Elements in parentheses are those responsible for the retroposition of the respective SINES. Green: tRNA-related sequence, beige: CORE sequence, dark grey: LINE3-like tail sequence, purple: RTE-like tail sequence, pink: origin unknown of the tail sequence, black: 7-nt insertion, red: 11-nt insertion, red AAAA: indicates that the SINE uses a poly(A)-tail for propagation via LINE1.

Comparative indel analysis of 75,000 full-length WSINE1s showed that there are two over-represented insertions. One is located at position 80 and the other at position 120 (Figure 3). It has been shown that the insert at position 80 is most likely the result of a duplication [30]. Alignments of the tRNA-related parts from several CORE-SINEs, both young and old, indicate that insertions and deletions are very infrequent in this region. No deletions were observed. Insertions were found in only three other CORE-SINEs (Mar1a_Mdo, MAR1, and WALLSI2) (data not shown). WSINE1 has no inserts, and can be considered ancestral to both WSINE1a (11-nt and 7-nt inserts) and WSINE1b (7-nt insert) (Figure 5). After screening the different marsupial SINEs, it is evident that the distinguishing 7-nt-long insert found in WSINE1a and WSINE1b is also present in WALLSI2 [30]. Insertions in the CORE-SINE tRNA region appear to be highly infrequent, therefore the presence of a 7-nt insert in a homologous position in WALLSI2 and WSINE1a/b indicates a common evolutionary ancestry. WSINE1a and WSINE1b share the 7-nt insert but 1a has received an additional 11-nt insert. Thus the acquisition of the 11-nt insert in WSINE1a would suggest an origin after 1b.

It was proposed that WALLSI2 (Mac1) might have originated from a WSINE1 (Mar3a), followed by the addition of a wallaby-specific tail [30]. However, the tail-region of WALLSI2 is shared by WALLSI1 and WALLSI3 and is therefore not wallaby-specific. The presence of the tail-region from WALLSI2 (in the elements WALLSI1 and WALLSI3) as well as WSINE1b in the Tasmanian devil, but absence of WALLSI2, suggest that the recombination into WALLSI2 must have happened after the split leading to Dasyuromorphia and occured only in Diprotodontia (wallaby). Molecular sequence estimates place the divergence between the two orders around 55–60 Myr ago [32,33,48].

The distribution of the three subtypes of WSINE1s differs among the Australian marsupial orders. In the Tasmanian devil, the 1b subtype is the most widespread, making up around 90% of the WSINE copies in the genome (Table 2); *Isoodon macrourus*, which belongs to the order Peramelemorphia, has a similar distribution of WSINE1s, with WSINE1b being dominant (Table 2) and WSINE1a being present in only very low copy numbers in both Dasyuromorphia and Peramelemorphia. Thus, the distribution of WSINE1b is consistent with the close relationship between Peramelemorphia and Dasyuromorphia [32,33,35].

### WSINE1 subfamilies and the evolution of Dasyuromorphia

SINEs propagate using one (or several) active master source copies, which produce copies that randomly integrate into the genome [49]. Over time, there have been several master copies within given SINE subtypes. In the human genome, the Alu SINEs seem to have had at least 143 active master copies during the 60 Myr that Alus were propagating [44]. By comparison, the platypus genome has had around 8 active LINE2 copies [29] that seem to have experienced a more linear evolution, where one master becomes inactivated and is then followed by a new one.

To investigate the network of WSINE1 subfamilies in the Tasmanian devil genome, we applied the same strategy used to estimate the segregation of Alu elements [44]. In total, 66 different WSINE1 subfamilies were identified from the 171,000 copies that aligned well to the consensus sequence (Figure 4Additional file 1: Table S3). A preliminary evolutionary age estimate of the different WSINE1 subfamilies was calculated using a local substitution rate of 0.0045/Myr, a value that was derived from WSINE1 insertions in the marsupial genome. Other substitution rates, derived from retroposons in general, L1 elements, and pseudogenes [50-52], were also applied to estimate the age of the retroposons. However, all these were too slow to estimate the age of WSINE1, because the resulting divergence time estimates predate the origin of Mammalia, and are thus highly incompatible with WSINE1 activity.

WSINE1 seems to have originated around 73 Myr ago, which corresponds on a marsupial evolutionary time scale to the split between the two South American orders Paucituberculata and Microbiotheria [32]. Although no data is currently available for Paucituberculata, WSINE1 elements were identified in the genome of Microbiotheria [33], but to date there is no evidence of WSINE1 elements in the genome of Didelphimorphia [9,11]. Thus, WSINE1s must have evolved after the split from Didelphimorphia (Opossum), either in Paucituberculata or Microbiotheria.

It has been shown that retroposon activation is correlated with speciation [53-55]. The network of SINE subfamilies coupled with the evolutionary age estimates and by correlation suggest that there was a burst of WSINE1 activity around the time of the origin of the order Dasyuromorphia (Figure 4), during which numerous speciation events lead to today’s dasyuromorphian families. The subfamily network also indicates that several WSINE1s became active master copies during the early phases of the order Dasyuromorphia; however, most were inactivated relatively quickly, leaving only one line of WSINE1 that continued producing copies until the present.

### CORE-SINEs in marsupials

CORE-SINEs belong to one of four super-classes of SINEs defined by highly conserved sequence domains [24,27], the others being V-SINEs [56], DeuSINEs [57,58] and CephSINEs [59]. The CORE-SINEs are tRNA-related, but their sequences have evolved beyond recognition [26], making it impossible to determine from which tRNA the CORE-SINE originated. Recently, a SINE from the insect Tobacco budworm (*Heliothis virescens*) was found to have a highly conserved, 65-nt-long CORE sequence [60].

Due to their high degree of conservation and abundance in mammals CORE-SINEs are speculated to have played, or still play, a functional role. Evidence for this hypothesis comes from one CORE-SINE that has been exapted into a neural enhancer [61].

The CORE-SINEs have been proliferating in marsupial genomes for at least 70 Myr. Several different CORE-SINEs, both young and old, are found in marsupials, but with different tail-regions depending on which LINE is responsible for their propagation [24,27,62]. Our genome analyses showed that the diagnostic 65-nt-long CORE sequence was truncated to 41 nt in a few of the marsupial CORE-SINEs (Figure 5) [30]. The WALLSI4, Mar1c_Mdo, WALLSI2, WSINE1, and SINE1_Mdo have shorter COREs, while Mar1a, b, MAR1, and all MIRs have the original CORE sequence. This implies that there are two types of CORE-SINEs in marsupial genomes, one with a short and one with a long CORE region. The short CORE-SINEs most likely have a shared evolutionary origin, and based on the TinT analysis, the oldest element with a short CORE is the WALLSI4. Both WSINE1 and SINE1_Mdo probably originated from the WALLSI4 or Mar1c_Mdo elements. WSINE1b and WALLSI2 both share the 7-nt-long insert in the tRNA-region, which indicates a close relationship between these elements, and that WALLSI2 evolved from WSINE1b. As WSINE1 is absent from the opossum genome, this in turn suggests that the truncation of the CORE must have happened independently in the two elements. Alternatively, they may have an origin in the same SINE family, such as WALLSI4 or Mar1c_Mdo. In the opossum genome, 600,000 copies of the SINE1_Mdo element make up the dominant SINE type [10], while in the wallaby, the WALLSI2, with about 48,000 copies, is the most recently active SINE [38]. In the Tasmanian devil genome, the WSINE1 had the most recent expansion and is present in 200,000 copies, making up 21 MB of the sequence. Thus, the activity and proliferation of the short CORE-SINEs in marsupials is an ongoing and successful process. In monotremes, another CORE-SINE, the MON, is active [29,63]. In placental mammals, the MIR and MIR3 are the only CORE-SINEs found, and these have been inactive for 130 Myr [26]. Thus, during the evolution to placental mammals, CORE-SINEs were inactivated. The activation and inactivation of SINEs and LINEs happens frequently in genomes over evolutionary times.

## Conclusions

The CORE-SINEs, a group of retroposons, is extinct in placental mammals since 150 Myr, while still proliferating in marsupials. We show that CORE-SINEs are the most recently active SINEs in the genome of the Tasmanian devil, and have since at least 60 Myr proliferated into nearly 200,000 copies. For the first time we have shown how an ancient speciation event in marsupials might have lead to a burst of retroposon activity that was partially silenced by the genome over time.

## Methods

### Whole genome screening

The genome of the Tasmanian devil [8] was screened for transposable elements using RepeatMasker 3.2.9 (http://www.repeatmasker.org) with the Repbase repeat libraries. Identified elements of different repeat element families were counted and summarized in Additional file 1: Table S1. Numerous marsupial SINEs are deposited in repeat libraries under different names while their consensus sequences are nearly identical. WALLSI3 is 99% similar to RTESINE2 and the same with WALLSI4 is 99% similar to WALLSI4_Mar. Therefore, in this study, only the WALLSI3 and WALLSI4 names and sequences are used. Correspondingly, the ancient mammalian interspersed repeats (MIR) are deposited under several names with nearly identical consensus sequences. We have used the names MIR/THER-1 (MIR, MIRb, MIRc, MIR_Mars, THER1_MD) and MIR3/THER-2 (MIR3, MIR3_MarsA and MIR3_MarsB) [24,26,27]. RepeatModeler (1.0.5) [64] was run to identify potential novel repeats in the Tasmanian devil genome.

### Transposition in Transposition

After conducting the full genome screening, we used the transposition in transposition (TinT) method [34] to estimate the temporal history of activity for the most abundant marsupial-specific SINEs and non-autonomous LINEs. The online program (http://www.compgen.uni-muenster.de/tools/tint/) uses the output from Repeatmasker, counts the number of nested transpositions, and estimates a relative time frame of activity for the selected types of SINEs ( Additional file 1: Table S2). This method is useful when searching for phylogenetically informative retroposons and for comparative genome studies.

### WSINE1 screening

The WSINE1 element was studied in further detail using a custom approach for identifying commonly occurring insertions/deletions (indels), implemented in a perl script (available on request). Pairwise alignments between all individual WSINE1 elements and each of the RepBase consensus sequences for WSINE1 and WSINE1a were constructed using the Needleman-Wunsch algorithm as implemented in the EMBOSS program needle [65]. For each position in the alignment, elements with an insertion or deletion in that specific position were counted and a plot showing the frequency of indels along the length of the element was created (Figure 3). This clearly showed indels that were overrepresented in the elements, allowing them to be further studied to identify previously unknown repeat families.

### WSINE1 distribution in Wallaby and Isoodon

To compare the distributions of the three subtypes of WSINE1 in two other orders of Australian marsupials, the genome of *Macropus eugenii* (Diprotodontia) and trace sequences of *Isoodon macrourus* (Peramelemorphia) were screened for the occurrence of WSINE1s using RepeatMasker 3.2.9 (http://www.repeatmasker.org) with the Repbase repeat libraries. All elements of full or almost full length (>130 bp) were selected and aligned to the WSINE1 consensus sequence. The alignments were checked for the previously identified signature indels and classified into one of the three WSINE1 types.

### Phylogeny and divergence time estimation

The complete mitochondrial genomes of six new dasyuromorphians, Tasmanian devil [GenBank: FN666604], planigale sp. [GenBank: FN666602], yellow-footed antechinus [GenBank: FN666600], numbat [GenBank: FN666603], dibbler [GenBank: FN666601], and the western quoll [GenBank: FN666605], were sequenced and analyzed at the nucleotide level (GTR + 4G + I), including all three codon positions, and amino acid (mtMam + 4G + I) levels using TreeFinder [66] ( Additional file 1: Table S7 and Additional file 1: Supplementary Methods). Alternative hypotheses were evaluated using S-H and AU tests ( Additional file 1: Table S5). Two different sets of fossil calibration points were used to estimate the divergence times of Dasyuromorphia, one set of 8 originating from [67] and one set of 11 taken from [33] ( Additional file 1: Table S8). Finally, the data were analyzed using a combination of the two sets of calibration points ( Additional file 1: Table S6 and Figure S2). The analyzed nucleotide data set is available in TREEBASE S12501.

### Evolutionary rate of WSINE1

Three WSINE1 insertions were previously found at narrowly defined evolutionary splits among marsupials [35]. When a SINE inserts *between* two evolutionary splits, it is impossible to say when during the time span the SINE inserted. Therefore, despite having several phylogenetically informative WSINE1 insertions in the marsupial phylogeny, only these three were deemed appropriate to estimate the substitution rate, as they had inserted during a relatively short time span and the evolutionary divergence estimates needed to calibrate the rate were uncontroversial ( Additional file 1: Table S4). An average HKY + G + I distance of the WSINE1 sequence insertions, excluding target site duplications and gaps, was calculated using TreeFinder [6]. The average distance values were divided by the upper and lower age of the split for all three insertions ( Additional file 1: Table S4).

### COSEG analysis

The COSEG program, which categorizes elements into subfamilies and creates a network-like distribution of a chosen class of retroposons, was applied to the WSINE1s [44]. The minimum number of elements required for a subfamily was set to 500 after testing different values ( Additional file 1: Table S5 and Additional file 1: Figure S2). As WSINE1 is a CORE-SINE, the results were closely screened to remove other CORE-SINEs included by mistake.

## Competing interests

The authors declare that they have no competing interests.

## Authors’ contributions

MN and BH designed the study. BH performed all bioinformatical screening. MN and BH interpreted the data. AJ provided mt sequence data and analyses. EPM and ZN coordinated genome sequencing, assembly and annotation. MN wrote the manuscript, and all co-authors approved and commented the manuscript. All authors read and approved the final manuscript.

## Supplementary Material

Additional file 1 **Table S1**. The numbers of SINEs, LINEs, and DNA transposons in the Tasmanian devil genome. **Table S2.** TinT matrix. **Table S3.** COSEG distance and count for 66 WSINE1 subfamilies. **Table S4.** Substitution rate estimation of WSINE and divergence times of splits. **Table S5.** ML analyses of alternative relationships inside Dasyuromorphia. **Table S6**. Divergence time estimates. **Table S7**. Marsupialian systematics and accession number of complete mt genomes. **Table S8**. Calibration points. **Figure S1.** Figure of the 66 WSINE1 sub-families in the Tasmanian Devil genome. **Figure S2**. Chronogram of marsupialian and placental mammalian divergences based on amino acid sequences and the Benton et al. 2009 calibration points. The numbers indicate the nodes given in table S4. Cret: Cretaceous, Pal: Paleocene, Eoc: Eocene, Oli: Oligocene, Mio: Miocene, P: Pliocene. **Supplementary methods. Supplementary results. Supplementary References**[68-77]. Click here for file
