# Supplementary material for: Expansion of CORE-SINEs in the genome of the Tasmanian devil
Source: BMC Genomics. 2012 May 6;13:172. doi: 10.1186/1471-2164-13-172 (PMC3403934; doi:10.1186/1471-2164-13-172)
Supplement: Additional file 1 — Table S1. The numbers of SINEs, LINEs, and DNA transposons in the Tasmanian devil genome. Table S2. TinT matrix. Table S3. COSEG distance and count for 66 WSINE1 subfamilies. Table S4. Substitution rate estimation of WSINE and divergence times of splits. Table S5. ML analyses of alternative relationships inside Dasyuromorphia. Table S6. Divergence time estimates. Table S7. Marsupialian systematics and accession number of complete mt genomes. Table S8. Calibration points. Figure S1. Figure of the 66 WSINE1 sub-families in the Tasmanian Devil genome. Figure S2. Chronogram of marsupialian and placental mammalian divergences based on amino acid sequences and the Benton et al. 2009 calibration points. The numbers indicate the nodes given in table S4. Cret: Cretaceous, Pal: Paleocene, Eoc: Eocene, Oli: Oligocene, Mio: Miocene, P: Pliocene. Supplementary methods. Supplementary results. Supplementary References[68-77]. [file 1471-2164-13-172-S1.doc]

Additional File 1

Expansion of CORE-SINEs in the genome of the Tasmanian Devil.

Nilsson, MA, Janke, A, Murchison, E, Ning, Z, Hallström, BM.

**Table S1.** The number of SINEs, LINEs and DNA transposons in the Tasmanian Devil genome.

**Table S2.** TinT matrix.

**Table S3.** COSEG distance and count for 66 WSINE1 sub-families.

**Table S4.** The substitution rate estimation of the WSINE1 found at different splits in the marsupial tree. a) Substitution Rate Estimation of WSINE1. b) Different divergence times of the nodes from mt and nuclear data.

**Table S5.** ML analyses of alternative relationships inside Dasyuromorphia.

**Table S6**. Divergence time estimates.

**Table S7**. Marsupialian systematics and accession number of complete mt genomes.

**Table S8**. Calibration points.

**Figure S1.** Figure of the 66 WSINE1 sub-families in the Tasmanian Devil genome.

**Figure S2**. Chronogram of marsupialian and placental mammal divergences based on aa sequences and the Benton et al. 2009 calibration points. The numbers indicate the nodes given in table S4. Cret: Cretaceous, Pal: Palaeocene, Eoc: Eocene, Oli: Oligocene, Mio: Miocene, P: Pliocene.

Supplementary methods

Supplementary results

Supplementary References [68-77]

**Table S1.** The total amount of transposable elements in the Tasmanian devil genome.

|  | **Number** | **Total nts** | **% of**  **genome** | **Opossum** | **Wallaby** |
| --- | --- | --- | --- | --- | --- |
| LINEs | **3208902** | **998820415** | **33.96%** | 29.17% | **28.6%** |
| SINEs | **2429154** | **351198812** | **10.89%** | 10.44% | **11.7%** |
| LTRs | **243978** | **52042725** | **1.72%** | 10.63% | **3.9%** |
| DNA transposons | **235659** | **33000300** | **1.13%** | 1.74% | **2.9%** |
| **Total** |  |  | **52.18%** | 52.17% | **52.8%** |

**Table S2**. Tint Matrix of frequently occurring SINEs and other short retroposons.

|  | MAR1 | MAR1a_Mdo | MAR1b_Mdo | MAR1c_Mdo | MIR | MIR3 | MdoRep1 | P7SL_MD | WALLSI1 | WALLSI1A | WALLSI3 | WALLSI4 | WSINE1 | WSINE1  a+b | [Sum] | Count | Avgsize | T(i) |
| --- | --- | --- | --- | --- | --- | --- | --- | --- | --- | --- | --- | --- | --- | --- | --- | --- | --- | --- |
| MAR1 | 1 | 7 | 5 | 2 | 59 | 37 | 11 | 2 | 0 | 1 | 3 | 10 | 4 | 1 | 143 | 22583 | 109,7 | 2615492 |
| MAR1a_Mdo | 20 | 26 | 116 | 37 | 1014 | 776 | 177 | 22 | 0 | 5 | 9 | 219 | 7 | 2 | 2430 | 138016 | 182,3 | 2722107 |
| MAR1b_Mdo | 10 | 22 | 51 | 21 | 978 | 515 | 112 | 26 | 0 | 7 | 7 | 177 | 7 | 1 | 1934 | 178996 | 152,1 | 2622872 |
| MAR1c_Mdo | 3 | 4 | 14 | 10 | 218 | 116 | 46 | 4 | 0 | 3 | 1 | 33 | 1 | 1 | 454 | 37453 | 135,3 | 2592694 |
| MIR | 20 | 20 | 93 | 25 | 1059 | 1499 | 143 | 22 | 0 | 18 | 9 | 280 | 7 | 4 | 3199 | 568828 | 116,8 | 1902451 |
| MIR3 | 4 | 8 | 5 | 1 | 1113 | 899 | 31 | 1 | 1 | 18 | 4 | 87 | 2 | 0 | 2174 | 641663 | 122,2 | 1668324 |
| MdoRep1 | 1 | 3 | 4 | 1 | 143 | 176 | 50 | 5 | 0 | 3 | 0 | 51 | 1 | 0 | 438 | 103721 | 132,5 | 2300632 |
| P7SL_MD | 0 | 0 | 0 | 0 | 20 | 20 | 3 | 1 | 0 | 0 | 1 | 10 | 0 | 0 | 55 | 12340 | 200,1 | 2410323 |
| WALLSI1 | 1 | 0 | 0 | 0 | 1 | 1 | 0 | 0 | 0 | 2 | 0 | 0 | 0 | 0 | 5 | 669 | 55,7 | 2663089 |
| WALLSI1A | 10 | 34 | 10 | 15 | 81 | 63 | 42 | 6 | 2 | 66 | 25 | 33 | 2 | 0 | 389 | 45349 | 233,5 | 2663621 |
| WALLSI3 | 5 | 16 | 17 | 18 | 112 | 96 | 51 | 5 | 0 | 9 | 56 | 37 | 0 | 0 | 422 | 33260 | 257,6 | 2634668 |
| WALLSI4 | 2 | 2 | 2 | 1 | 314 | 125 | 23 | 3 | 0 | 6 | 1 | 17 | 2 | 0 | 498 | 131879 | 151,3 | 2193234 |
| WSINE1 | 38 | 140 | 152 | 37 | 561 | 590 | 123 | 14 | 1 | 106 | 59 | 241 | 11 | 11 | 2084 | 87340 | 125,9 | 2933083 |
| WSINE1  a+b | 368 | 611 | 1024 | 102 | 995 | 284 | 88 | 13 | 0 | 91 | 33 | 86 | 3 | 5 | 3703 | 122529 | 134 | 3152414 |
| [Sum] | 483 | 893 | 1493 | 270 | 6668 | 5197 | 900 | 124 | 4 | 335 | 208 | 1281 | 47 | 25 | 0 | 2124626 | 0 | null |

**Table S3.** The distance and total count value from each of the 66 sub-families identified by COSEG.

| **SUBFAMILY** | **Transversion**  **distance** | **Uncorrected distance** | **COSEQ distance** | **Number of copies** |
| --- | --- | --- | --- | --- |
| **1** | 0,066 | 0,189 | 0,269 | 9727 |
| **2** | 0,084 | 0,216 | 0,32 | 784 |
| **3** | 0,080 | 0,221 | 0,305 | 3304 |
| **4** | 0,055 | 0,168 | 0,225 | 4106 |
| **5** | 0,081 | 0,220 | 0,305 | 1613 |
| **6** | 0,064 | 0,193 | 0,254 | 6037 |
| **7** | 0,101 | 0,249 | 0,342 | 2760 |
| **8** | 0,074 | 0,209 | 0,301 | 2949 |
| **9** | 0,058 | 0,179 | 0,238 | 4796 |
| **10** | 0,033 | 0,112 | 0,151 | 2704 |
| **11** | 0,086 | 0,227 | 0,321 | 3246 |
| **12** | 0,062 | 0,189 | 0,25 | 6953 |
| **13** | 0,081 | 0,220 | 0,311 | 3769 |
| **14** | 0,065 | 0,194 | 0,258 | 2701 |
| **15** | 0,110 | 0,262 | 0,355 | 1451 |
| **16** | 0,113 | 0,289 | 0,36 | 1110 |
| **17** | 0,082 | 0,288 | 0,306 | 1385 |
| **18** | 0,084 | 0,284 | 0,286 | 571 |
| **19** | 0,101 | 0,294 | 0,336 | 1864 |
| **20** | 0,099 | 0,282 | 0,338 | 1934 |
| **21** | 0,091 | 0,279 | 0,321 | 1155 |
| **22** | 0,067 | 0,260 | 0,282 | 1415 |
| **23** | 0,061 | 0,245 | 0,251 | 7885 |
| **24** | 0,062 | 0,253 | 0,255 | 7297 |
| **25** | 0,072 | 0,273 | 0,296 | 5636 |
| **26** | 0,064 | 0,258 | 0,249 | 7037 |
| **27** | 0,059 | 0,248 | 0,231 | 7446 |
| **28** | 0,066 | 0,264 | 0,263 | 4582 |
| **29** | 0,065 | 0,265 | 0,268 | 1381 |
| **30** | 0,068 | 0,265 | 0,273 | 4023 |
| **31** | 0,068 | 0,255 | 0,276 | 1968 |
| **32** | 0,072 | 0,266 | 0,286 | 1323 |
| **33** | 0,065 | 0,242 | 0,273 | 3306 |
| **34** | 0,072 | 0,253 | 0,295 | 1616 |
| **35** | 0,046 | 0,207 | 0,211 | 2015 |
| **36** | 0,060 | 0,218 | 0,242 | 1191 |
| **37** | 0,066 | 0,245 | 0,282 | 778 |
| **38** | 0,080 | 0,259 | 0,312 | 576 |
| **39** | 0,063 | 0,264 | 0,26 | 1403 |
| **40** | 0,101 | 0,291 | 0,331 | 606 |
| **41** | 0,027 | 0,135 | 0,124 | 1507 |
| **42** | 0,064 | 0,252 | 0,256 | 2010 |
| **43** | 0,054 | 0,224 | 0,222 | 2438 |
| **44** | 0,071 | 0,275 | 0,287 | 500 |
| **45** | 0,058 | 0,242 | 0,235 | 2614 |
| **46** | 0,069 | 0,204 | 0,275 | 1913 |
| **47** | 0,086 | 0,237 | 0,321 | 674 |
| **48** | 0,067 | 0,194 | 0,264 | 875 |
| **49** | 0,081 | 0,223 | 0,27 | 993 |
| **50** | 0,068 | 0,195 | 0,26 | 5775 |
| **51** | 0,056 | 0,176 | 0,227 | 3445 |
| **52** | 0,066 | 0,189 | 0,263 | 4106 |
| **53** | 0,055 | 0,172 | 0,223 | 4426 |
| **54** | 0,058 | 0,178 | 0,234 | 568 |
| **55** | 0,053 | 0,168 | 0,22 | 710 |
| **56** | 0,078 | 0,213 | 0,307 | 998 |
| **57** | 0,070 | 0,207 | 0,275 | 659 |
| **58** | 0,060 | 0,184 | 0,243 | 1785 |
| **59** | 0,054 | 0,164 | 0,217 | 678 |
| **60** | 0,060 | 0,184 | 0,24 | 1400 |
| **61** | 0,054 | 0,171 | 0,223 | 1558 |
| **62** | 0,054 | 0,168 | 0,222 | 1397 |
| **63** | 0,058 | 0,181 | 0,239 | 628 |
| **64** | 0,060 | 0,185 | 0,244 | 717 |
| **65** | 0,055 | 0,168 | 0,22 | 1630 |
| **66** | 0,081 | 0,223 | 0,296 | 1368 |
| **Total** |  |  |  | 171775 |

**Table S4**. The substitution rate estimation of the WSINE1 found at different splits in the marsupial tree.

a) Substitution rate estimation of WSINE1.

|  | HKY distance | Oldest age | Youngest age |
| --- | --- | --- | --- |
| Node 1=129 | 0,354 | 72 my | 65 my |
| Australidelphia |  | 0,353/72= | 0,353/63= |
|  |  | **0,0049** | **0,0054** |
|  |  |  |  |
| Node 2= 206 | 0,285 | 61 my | 55 my |
| Diprotodontia |  | 0,285/61= | 0,285/55= |
|  |  | **0,0046** | **0,0051** |
|  |  |  |  |
| Node 3=194 | 0,217 | 53 my | 48 my |
| Phalangerida |  | 0,217/53= | 0,217/48= |
|  |  | **0,0041** | **0,0045** |
| Average subs/my |  | **0,0045** | **0,0050** |

b) Different divergence times of the nodes from mt and nuclear data.

|  | Meredith et al.  2009 |  | Meredith et al  2008 |  | This study |
| --- | --- | --- | --- | --- | --- |
| Split prior to Diprotodontia | 62,2 my |  | 59,2-62,8 my |  | 61 my |
|  |  |  |  |  |  |
| Origin of  Diprotodontia | 53,3 my (46,6-60) |  | 50,8-54,1 my |  | 55 my |
|  |  |  |  |  |  |
| Origin of  Phalangerida | 48,4 my  (42,1-55,1) |  | 43,8-47,6 my |  | 53 my |
|  |  |  |  |  |  |
| Origin of  Australidelphia | 63,0 my  55,6-70,0) |  | 59,9-62,9 my |  | 65 my |
|  |  |  |  |  |  |
| Split prior to Australidelphia | 72,5 my  (65,2-86,2) |  | 71,3-85,6 my |  | 72 my |

[32] Meredith RW, Westerman M, Case JA, Springer MS. 2008. A phylogeny and timescale for marsupial evolution based on sequences for five nuclear genes. *Journal of Mammalian Evolution* **15**:1-36.

[47] Meredith RW, Westerman M, Springer MS. 2009. A phylogeny of Diprotodontia (Marsupialia) based on sequences for five nuclear genes. *Mol Phylogenet Evol*. **51**:554-571.

**Table S5**. ML analyses of alternative relationships inside Dasyuromorphia.

| Topology | Shimodaira-Hasegawa test (SH) | | | Approximately unbiased test (AU) | | |
| --- | --- | --- | --- | --- | --- | --- |
|  | 12 cdp | 123 cdp | aa | 12 cdp | 123 cdp | aa |
| OG,(T,(D,M)) | 1.00 | 1.00 | 0.39 | 0.88 | 0.94 | 0.32 |
| OG,(M,(D,T)) | 0.07 | 0.02* | 0.11 | 0.06 | 0.01* | 0.05 |
| OG,(D,(T,M)) | 0.21 | 0.07 | 1.00 | 0.16 | 0.09 | 0.73 |
| OG,(S,(H,G)) | 1.00 | 1.0 | 0.53 | 0.79 | 0.98 | 0.42 |
| OG,(G,(S,H)) | 0.40 | 0.00* | 0.55 | 0.32 | 0.00* | 0.46 |
| OG,(H,(S,G)) | 0.31 | 0.0* | 1.00 | 0.24 | 0.00* | 0.66 |

Note - A star indicates *hypotheses that are rejected at the 5% level of significance.* OG: Outgroup; D: Dasyuridae; M: Myrmecobiidae; T: Thylacinidae. S : Tasmanian devil; G: western quoll; H: northern quoll.

**Table S6**. Divergence time estimates in Ma using [67] and [32] and one analysis combining the calibration points from both studies. For branch numbering refer to figure S2. n.a. - Not applicable.

| Branch | Benton et al. 2009 | Meredith et al. 2008 | Combined  set |
| --- | --- | --- | --- |
| 1 | 50 | n.a. | 50 |
| 2 | 71 | n.a. | 71 |
| 3 | 43 | n.a. | 43 |
| 4 | 95 | n.a. | 95 |
| 5 | 65 | n.a. | 65 |
| 6 | 105 | n.a. | 105 |
| 7 | 138 | n.a. | 138 |
| 8 | 25 | 32 | 25 |
| 9 | 80 | 75 | 80 |
| 10 | 51 | 50 | 51 |
| 11 | 42 | 43 | 42 |
| 12 | 6 | 5 | 6 |
| 13 | 71 | 68 | 72 |
| 14 | 9 | 8 | 8 |
| 15 | 25 | 21 | 23 |
| 16 | 37 | 31 | 34 |
| 17 | 66 | 60 | 65 |
| 18 | 62 | 56 | 61 |
| 19 | 56 | 51 | 55 |
| 20 | 44 | 40 | 44 |
| 21 | 53 | 48 | 53 |
| 22 | 45 | 41 | 45 |
| 23 | 36 | 33 | 36 |
| 24 | 41 | 37 | 41 |
| 25 | 50 | 45 | 50 |
| 26 | 23 | 20 | 23 |
| 27 | 8 | 7 | 8 |
| 28 | 17 | 15 | 17 |
| 29 | 44 | 40 | 44 |
| 30 | 26 | 23 | 26 |
| 31 | 63 | 58 | 63 |
| 32 | 60 | 55 | 60 |
| 33 | 45 | 41 | 45 |
| 34 | 40 | 67 | 40 |
| 35 | 13 | 11 | 13 |
| 36 | 23 | 20 | 23 |
| 37 | 31 | 28 | 31 |
| 38 | 13 | 11 | 12 |
| 39 | 14 | 12 | 14 |
| 40 | 19 | 17 | 18 |
| 41 | 26 | 23 | 26 |
| 42 | 19 | 17 | 19 |

**Table S7.** Marsupialian systematics and accession number of complete mt genomes used in this study.

| **Infraclass – Marsupialia** | |
| --- | --- |
| **Cohort – Australidelphia** | |
| **Order – Dasyuromorphia** | |
| **Family – Dasyuridae** | |
| **Subfamily – Dasyurinae** | |
| **Tribe – Dasyurini** | |
| **Genus - *Parantechinus*** | |
|  | *Parantechinus apicalis* (dibbler, FN666601) |
| **Genus – *Dasyurus*** | |
|  | *Dasyurus geoffroii* (western quoll, FN666605) |
|  | *Dasyurus hallucatus* (northern quoll, AY795973) |
| **Genus – *Sarcophilus*** | |
|  | *Sarcophilus harrisii* (tasmanian devil, FN666604) |
| **Tribe – Phascogalini** | |
| **Genus – *Antechinus*** | |
|  | *Antechinus flavipes* (yellow-footed antechinus, FN666600) |
| **Genus – *Phascogale*** | |
|  | *Phascogale tapoatafa* (brush-tailed phascogale, AJ639869) |
| **Subfamily - Sminthopsinae** | |
| **Tribe – Planigalini** | |
| **Genus – *Planigale*** | |
|  | *Planigale sp.* (n/a, FN666602) |
| **Tribe – Sminthopsini** | |
| **Genus – *Sminthopsis*** | |
|  | *Sminthopsis crassicaudata* (fat-tailed dunnart, AY795974) |
|  | *Sminthopsis douglasi* (julia creek dunnart, AJ639867) |
| **Family – Myrmecobiidae** | |
|  | *Myrmecobius fasciatus* (numbat, FJ515782/ FN666603) |
| **Family – Thylacinidae** | |
|  | *Thylacinus cynocephalus* (tasmanian wolf, FJ515780) |
| **Order – Diprotodontia** | |
|  | *Distoechurus pennatus* (feather-tailed possum, AB241052) |
|  | *Lagorchestes hirsutus* (rufous hare-wallaby, AB241056) |
|  | *Lagostrophus fasciatus* (banded hare wallaby, AM262148) |
|  | *Macropus robustus* (common wallaroo, Y10524) |
|  | *Petaurus breviceps* (sugar glider, AB241055) |
|  | *Dactylopsila trivirgata* (striped possum, AB241054) |
|  | *Phalanger interpositus* (stein's cuscus, AB241057) |
|  | *Trichosurus vulpecula* (common brushtail possum, AF357238) |
|  | *Phascolarctos cinereus* (koala, AB241053) |
|  | *Potorous tridactylus* (long-nosed potoroo, AJ639873) |
|  | *Pseudocheirus peregrinus* (common ringtail possum, AJ639870) |
|  | *Tarsipes rostratus* (honey possum, AJ639868) |
|  | *Vombatus ursinus* (common wombat, AJ304826) |
| **Order – Microbiotheria** | |
|  | *Dromiciops gliroides* (monito del monte, AJ508402) |
| **Order – Notoryctemorphia** | |
|  | *Notoryctes typhlops* (southern marsupial mole, AJ639874) |
| **Order – Peramelemorphia** | |
|  | *Isoodon macrourus* (northern brown bandicoot, AF358864) |
|  | *Perameles gunnii* (eastern barred bandicoot, AJ639872) |
|  | *Echymipera rufescens australis* (long-nosed spiny bandicoot, AY795975) |
|  | *Macrotis lagotis* (greater bilby, AJ639871) |
| **Cohort – Ameridelphia** | |
| **Order – Didelphimorphia** | |
|  | *Didelphis virginiana* (north american opossum, Z29573) |
|  | *Metachirus nudicaudatus* (brown four-eyed opossum, AJ639866) |
|  | *Monodelphis domestica* (gray short-tailed opossum, AJ508498) |
|  | *Thylamys elegans* (elegant fat-tailed mouse opossum, AJ508401) |
| **Order – Paucituberculata** | |
|  | *Caenolestes fuliginosus* (silky shrew opossum, AJ508400) |
|  | *Rhyncholestes raphanurus* (long-nosed shrew opossum, AJ508399) |
| **Infraclass – Eutheria** | |
|  | *Bos Taurus* (cow, J01394) |
|  | *Canis familiaris* (dog, U96639) |
|  | *Ceratotherium simum* (white rhinoceros, Y07726) |
|  | *Dasypus novemcinctus* (nine-banded armadillo, Y11832) |
|  | *Equus caballus* (horse, X79547) |
|  | *Felis catus* (cat, U20753) |
|  | *Balaenoptera musculus* (blue whale, X72204) |
| **Infraclass – Monotremata** | |
|  | *Ornithorhynchus anatinus* (duck-billed platypus, X83427) |

**Table S8.** **Calibration points.**

The two sets of calibration points used in the estimation of dasyuromorphian divergence times. Calibration points have been collected from [67] and [32]. ^a^ and ^b^ dates are taken from phylogenomic analyses [68].

|  | Benton et al. 2009 | Meredith et al. 2008 |
| --- | --- | --- |
| Fixed point | Node 7: 138^a^ Ma | Node 9: 75 Ma |
|  | Node 1: 50-60^b^ Ma | Node 10: 7-56 Ma |
|  | Node 2: 63-132 Ma | Node14: 4-23 Ma |
|  | Node 3: 40-65 Ma | Node 15: 4-23 Ma |
|  | Node 5: 52-66 Ma | Node 17: 55-71 Ma |
|  | Node 4: 63-132 Ma | Node 20: 26-65 Ma |
|  | Node 6: 63-132 Ma | Node 21: 26-55 Ma |
|  | Node 9: 62-132 Ma | Node 23: 26-65 Ma |
|  |  | Node 26: 12-34 Ma |
|  |  | Node 37: 4-34 Ma |
|  |  | Node 42: 4-23 Ma |

x


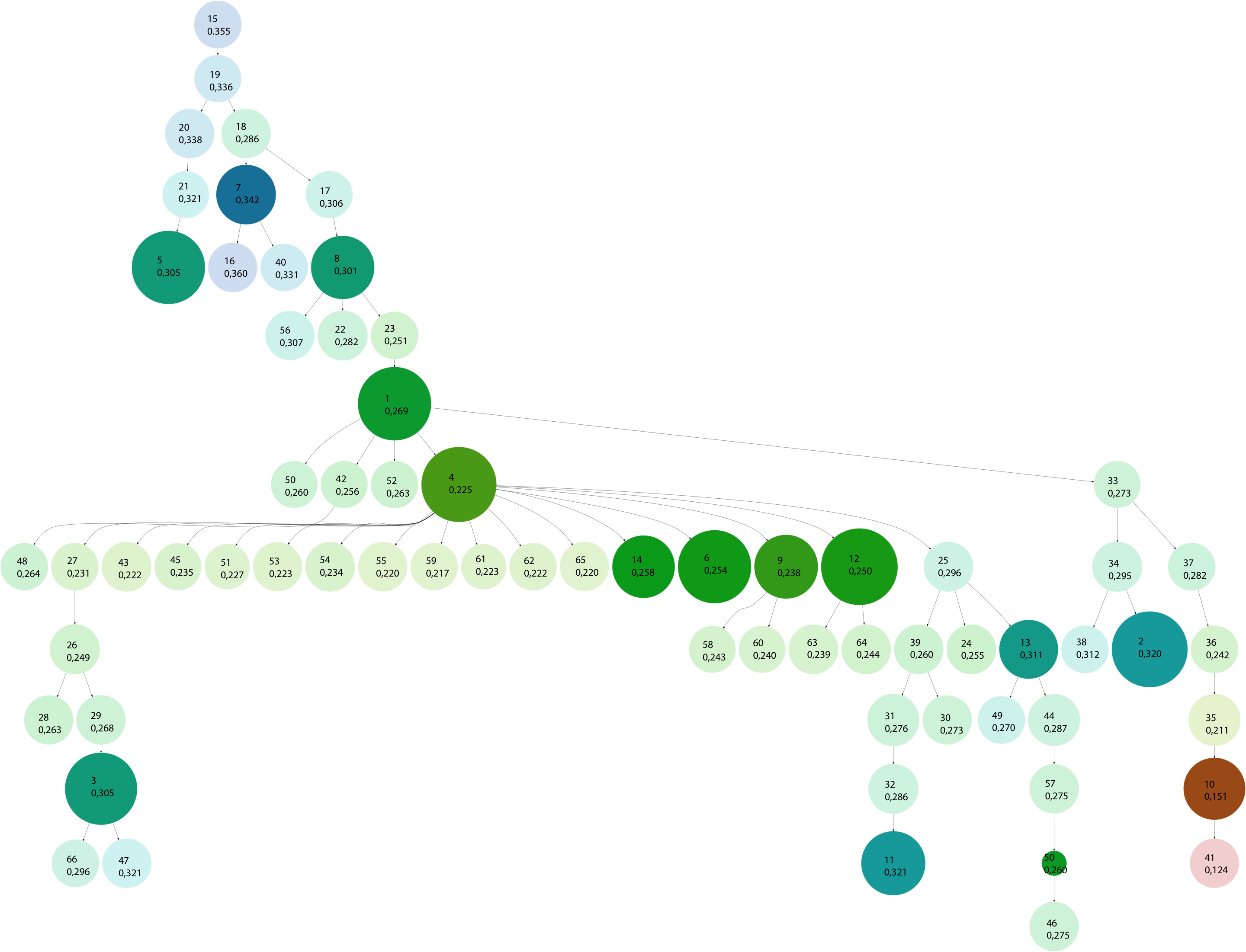


**Figure S1**. The network of 66 sub-families of WSINE1 in the Tasmanian Devil genome. The upper number in each ball indicates the sub-family name, and the value below the distance. For a total list of element count and divergence please see table S3.


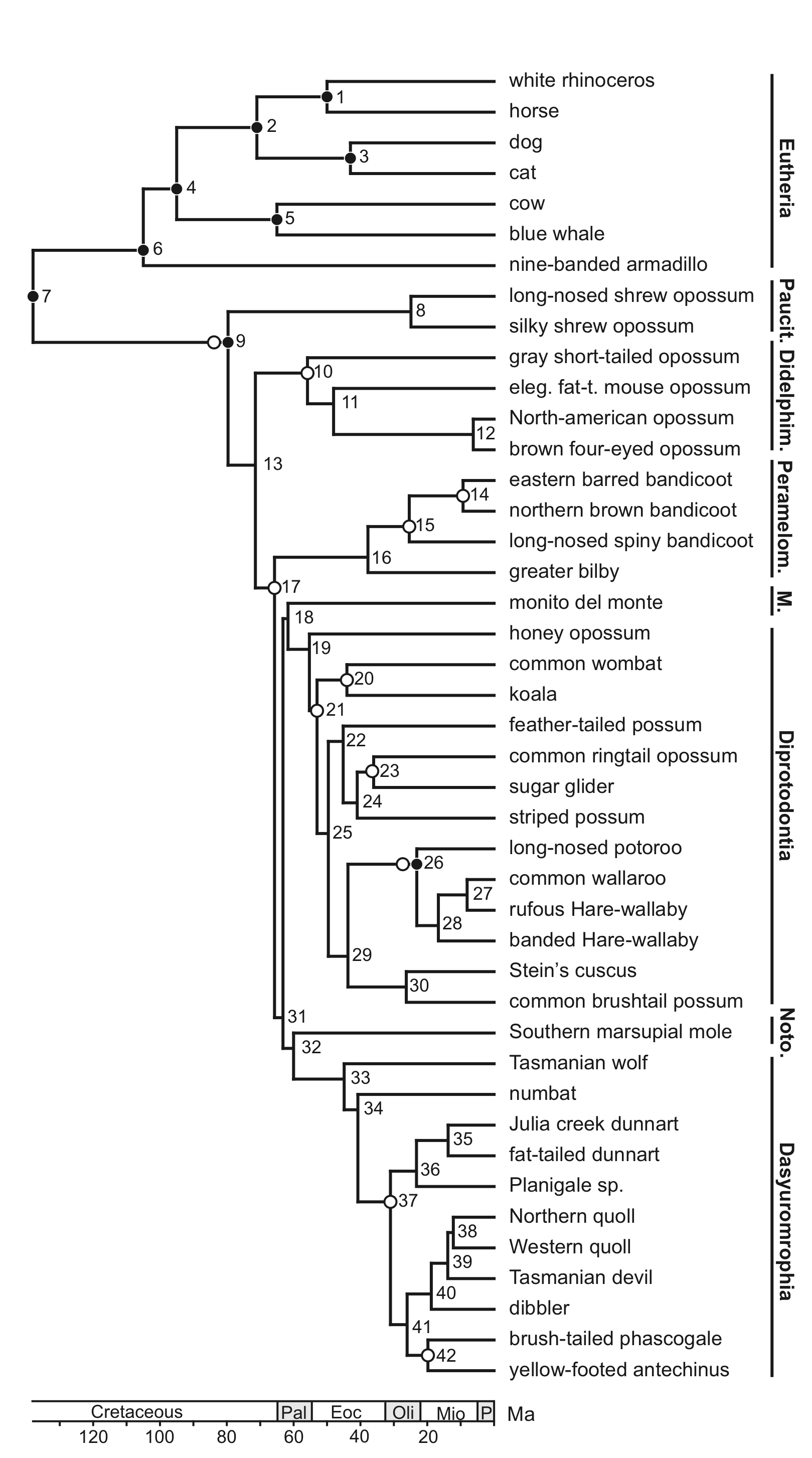


**Figure S2**. Chronogram based on the 44-taxon data set. For divergence times see table S6. Solid circles refer to calibration points of [67] and open to [32]. Paucit. – Paucituberculata, Didelphim. –Didelphimorphia, Peramelom. – Peramelomorphia, M. – Microbitheria, Noto. - Notoryctemorphia.

SUPPLEMENTARY METHODS

PHYLOGENETIC RECONSTRUCTION AND DIVERGENCE TIME ESTIMATION

_____________________________________________________________________

*DNA extraction, PCR amplification and sequencing*

Six dasyuromorphian species were sequenced, that of the dibbler, *Parantechinus apicalis*, the Tasmanian devil, *Sarcophilus harrisii*, the Western Quoll, *Dasyurus geoffroii*, the yellow-footed antechinus, *Antechinus flavipes,* an unspecified species of the genus *Planigale*, *Planigale sp*., and the Numbat *Myrmecobius fasciatus*. Total DNA was extracted from tissue samples using the phenol-chloroform method [69].

The *LA Taq,* *Z-Taq, or Ex Taq* (Takara Bio Inc.) DNA polymerases were used for amplification according to the manufacturer’s specifications. In cases with several amplification products, the correct band was gel eluted using the Gel Band Purification Kit (Pharmacia Biotech) prior to sequencing.

Most of the coding regions were unproblematic to amplify in fragments sizes up to 5 kilo bases (Kb). All fragments were overlapping by about 500 nucleotides (nt) and were sequenced from both strands when sequencing artifacts occurred or were suspected. The conserved PCR primers and numerous specific primers for primer walking were used for sequencing with the BigDye Terminator v3.1 Cycle Sequencing Kit (Applied Biosystems) according to the manufacturers recommendations. The reactions were analyzed on an ABI prism 3100 Genetic Analyzer.

*Data alignment and phylogenetic analyses*

The sequences were assembled manually in the program Se-Al [70]. Each protein-coding gene was translated for verifying the reading frame and for detection of sequencing artifacts. The alignment of the sequences was done manually in PAUP* [71] by adding the twelve H-strand protein-coding genes to an existing alignment of marsupialian and placental mammalian sequences [31]. Gaps and alignment ambiguous sites adjacent to the gaps were removed with the aid of a custom made PERL program.

Modeltest version 3.7 and Prottest version 1.2.6 were used for evaluating the best-fitting nt and amino acid (aa) models for the maximum likelihood (ML) analyses [72,73]. The ML phylogenetic analyses were done in TREEFINDER [66] (TF).

The nt data were analyzed by the general time reversible model of sequence evolution, GTR [74], GTR2 [66] assuming four classes of rate heterogeneity, 4G [75] and one class of invariable sites, I. The alignment was analyzed including all codon positions using the GTR+4G+I model. The aa sequences were analyzed using the mtMAM model of sequence evolution and 4G+I. TF branch support values were calculated and alternative topologies were evaluated by the Shimodaira-Hasegawa probability values [76] (pSH) and Approximately Unbiased probability values [77], pAU.

*Local calibration of evolutionary rates*

Three WSINE1 containing loci placed within narrowly defined divergences were used as calibration for the rate.

1. The sequence distance within the group of marsupials, excluding target site duplication, was calculated using HKY+G+I using Treefinder [66]. As the exact time of insertion can have occurred at any time between the upper and lower split, an average was done between oldest and youngest date. This gives an average rate of substitution per million years. This rate is specific for marsupials, and in particular for WSINE1.
2. Due to the limited number of loci and sequences, we have cross-compared all and these were found to correlate.
3. The evolutionary nodes in questions have been estimated by different data sets to the same age.
4. The divergence for each sub-family was estimated using the calculated rate by dividing the rate with the divergence.

SUPPLEMENTARY RESULTS

*Phylogenetic analysis*

***Dasyuromorphian phylogeny***

The 16 species dataset is 10,845 nucleotide (nt) sites (3,615 amino acid (aa) sites) long. A chi-2 test for compositional homogeneity on the complete alignment showed that the overall nucleotide composition differs significantly over the complete data set, but was homogenous for 1^st^ and 2^nd^ codon position (cdp) in marsupials for most species and for all species among the Dasyuromorphia. Recoding the sequences to R and Y increased the number of species that conform in compositional homogeneity. The aa composition was homogenous for most marsupials and all Dasyuromorphia. The programs Modeltest and Prottest suggested the GTR+4G+I model for ML analyses of the analysis of 1^st^+2^nd^ cdp and all cdp of nt sequence data and the mtMam+4G+I model for the analyses of aa sequences, respectively. Within the Dasyuridae most branches are maximally supported with TF values being 99% or better, except for the divergence between the genera *Dasyurus* (Quolls) and *Sarcophilus*, the Tasmanian devil (Supplementary Figure S2). Their relationship is differently resolved in the nt and aa sequence based analyses. While ML analysis of the aa data show weak support for *Sarcophilus* nested inside the quolls*,* the ML analyses of nt sequences strongly support at monophyletic genus *Dasyurus*. ML analysis of all three codon positions (123 cdp), clearly rejects that the genus *Sarcophilus* being nested inside *Dasyurus* by SH and AU test statistics (Supplementary Table S5).

***Dasyruromorphia divergence times***

The origin of the order Dasyuromorphia is calculated to 60/54.8 million years ago (Ma) based on [67], the first value, or [32] shown in the second value. The deepest split is between the Tasmanian wolf (Thylaciniiade) and the remaining Dasyuromorphia at 44.9/40.9 Ma. The next divergence occurred 3-4 my later (40.8/37.2 Ma) between numbat (Myrmecobiiade) and Dasyuridae. The deepest split inside Dasyuridae is estimated to 31/27.8 Ma between the two subfamilies Sminthopsinae and Dasyurinae. Within Sminthopsinae, the Planigalini (*Planigale sp*.) and Sminthopsini diverged at 23.2/20.4 Ma while closely related species within Sminthopsini diverged at 13.8/12.1 Ma. Inside Dasyurinae the tribe Phascogalini originated at 26/23.1 Ma. The species within Phascogalini split at 19.8/17.6 Ma. The genus *Parantechinus* within the tribe Dasyurini diverged from the two genera *Dasyurus* and *Sarcophilus* at 18.8/16.7 Ma (*Parantechinus apicalis*) and the later two genera diverge at 13.9/12.3 Ma (Tasmanian devil). The two closest related species in the genus *Dasyurus* diverge at 12.2/10.8 Ma. The overall similarity between the different calibration points is remarkable.
